# Phylogeny of the Archiborborinae (Diptera: Sphaeroceridae) Based on Combined Morphological and Molecular Analysis

**DOI:** 10.1371/journal.pone.0051190

**Published:** 2013-01-18

**Authors:** Joel H. Kits, Stephen A. Marshall, Jeffrey H. Skevington

**Affiliations:** 1 University of Guelph Insect Collection and Insect Systematics Laboratory, School of Environmental Sciences, University of Guelph, Guelph, Ontario, Canada; 2 Agriculture and Agri-Food Canada, Canadian National Collection of Insects, Arachnids and Nematodes, Ottawa, Ontario, Canada; 3 Department of Biology, Carleton University, Ottawa, Ontario, Canada; University of California, Berkeley, United States of America

## Abstract

The Archiborborinae is a diverse Neotropical subfamily of Sphaeroceridae, with many undescribed species. The existing generic classification includes three genera consisting of brachypterous species, with all other species placed in the genus *Archiborborus*. We present the first phylogenetic hypothesis for the subfamily based on morphological, molecular, and combined datasets. Morphological data include 53 characters and cover all valid described taxa (33 species in 4 genera) in the subfamily, as well as 83 undescribed species. Molecular data for five genes (mitochondrial 12S rDNA, cytochrome c oxidase subunit I, and cytochrome B, and nuclear alanyl-tRNA synthetase and 28S rDNA) were obtained for 21 ingroup taxa. Data support the separation of the Archiborborinae from the Copromyzinae, with which they were formerly combined. Analyses support consistent groups within the subfamily, but relationships between groups are poorly resolved. The validity of the brachypterous genera *Penola* Richards and *Frutillaria* Richards is supported. The former genus *Archiborborus* Duda is paraphyletic, and will be divided into monophyletic genera on the basis of this work. Aptery and brachyptery have evolved multiple times in the subfamily. *Antrops* Enderlein, previously including a single brachypterous species, is a senior synonym of *Archiborborus*.

## Introduction

The Archiborborinae is an entirely Neotropical clade of Sphaeroceridae, first recognized as a subfamily by Kits and Marshall [1]. The most recent classification of the subfamily [2] (as the tribe Archiborborini in the subfamily Copromyzinae) includes four genera: *Antrops* Enderlein (type: *Antrops truncipennis* Enderlein), *Archiborborus* Duda (type: *Archiborborus submaculatus* Duda ( = *Archiborborus femoralis* (Blanchard)), *Penola* Richards (type: *Penola eudyptidis* Richards), and *Frutillaria* Richards (type: *Frutillaria kuscheli* Richards). All except *Archiborborus* include only flightless species with highly reduced wings. The subfamily is speciose but poorly known; we recently described seven new species in the genus *Frutillaria* [1] and we will be describing approximately 80 new species of Archiborborinae in upcoming papers. Phylogenetic analysis is required to resolve two taxonomic problems relating to the subfamily: the relationships of the Archiborborinae to other Sphaeroceridae, and the generic classification of the subfamily.

The Archiborborinae were first treated as a group by Hackman [3] although Richards [4], [5] earlier acknowledged the relationship between his new genera *Penola* and *Frutillaria* and the genus *Archiborborus*. The group was treated as a tribe, Archiborborini, by Norrbom and Kim [6], who considered the included genera to form a clade sister to the Holarctic and Old World genera of the tribe Copromyzini. Although Norrbom and Kim included several archiborborines as outgroup taxa in their analysis of copromyzine relationships, they did not explicitly analyse whether the two tribes were in fact sister taxa. As well, none of these previous authors have attempted to resolve relationships within the subfamily in a cladistic analysis.

This analysis represents the first phylogenetic hypothesis for the Archiborborinae. The molecular analysis is also the first published for the Sphaeroceridae, although sphaerocerids have been included as outgroups in previous phylogenetic studies on other groups [7]–[9] and were included in the FLYTREE project [10]. Furthermore, with outgroups representing several major clades of Sphaeroceridae, this is the first study to provide quantitative evidence for subfamily-level phylogenetic relationships of the family. Although the resolution of our results is fairly low, we recover several groups consistently and provide limited data on their relationships.

## Materials and Methods

### Taxon sampling

**Figure 1:**
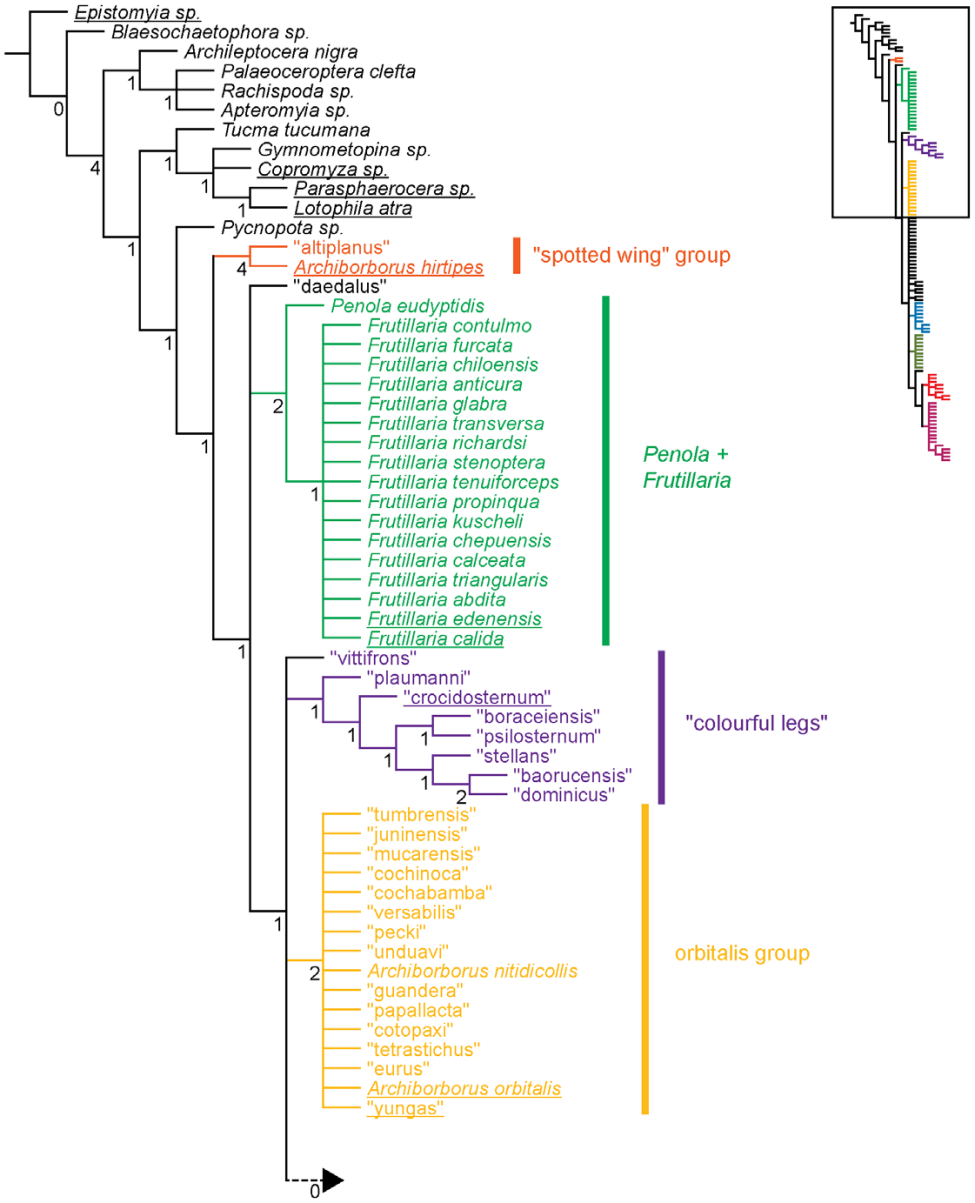
Parsimony analysis of morphological dataset. Strict consensus of 10000 trees, length 210. Numbers beneath nodes are Bremer support indices. Dashed branches (with Bremer support of 0) indicate clades only found in the consensus after pruning the taxa “apterus” and “biflavus”. Taxa with names underlined were used as exemplars in molecular analyses.

**Figure 2:**
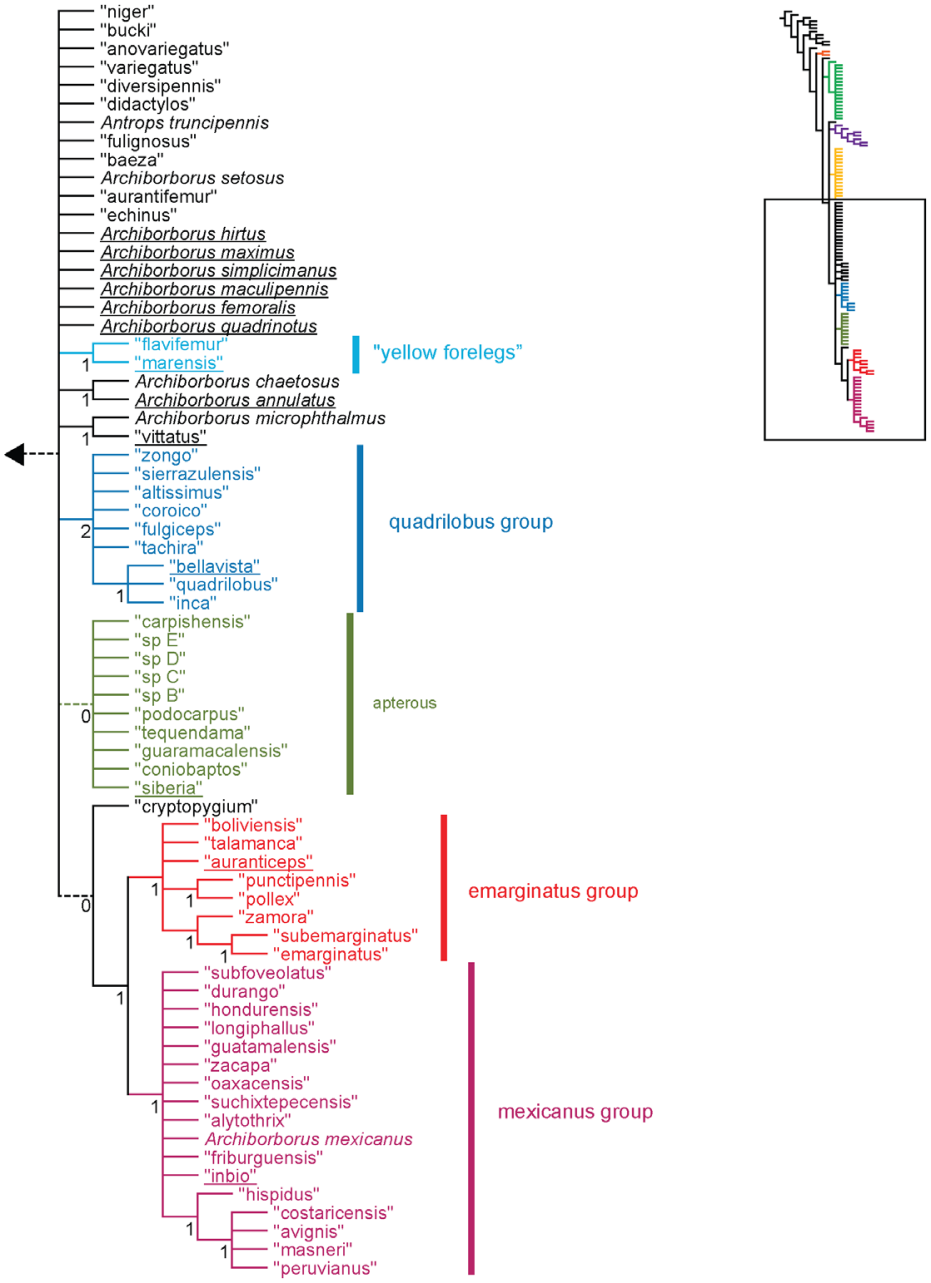
Parsimony analysis of morphological dataset, continued. Strict consensus of 10000 trees, length 210.. Numbers beneath nodes are Bremer support indices. Dashed branches (with Bremer support of 0) indicate clades only found in the consensus after pruning the taxa “apterus” and “biflavus”. Taxa with names underlined were used as exemplars in molecular analyses.

All 33 valid, described species of Archiborborinae, as well as 83 undescribed species, were included in the morphological matrix. Outgroups included multiple representatives of four of the five non-archiborborine sphaerocerid subfamilies. An undescribed species of the genus *Pycnopota*, which cannot be confidently placed in any known sphaerocerid subfamily, was also included. Non-sphaerocerid outgroups included two representatives of the heleomyzid subfamily Cnemospathidinae (*sensu* McAlpine [11]). The taxa selected for sequencing represented most of the clades identified in the morphological analysis (Figures 1, 2), as well as outgroups representing the Heleomyzidae, *Pycnopota*, and three other sphaerocerid subfamilies. Undescribed species are referred to in the text and figures with single names in quotations marks; these names refer to manuscript names and are not considered published under the rules of the ICZN.

### Morphological characters

The characters used for the morphological analysis include previously published characters thought to characterize the Archiborborinae or groups within the subfamily, as well a number of newly recognized characters. Some characters of broader significance within the Sphaeroceridae were included in an effort to resolve the position of the Archiborborinae within the family. The complete matrix is presented in Nexus S1.

*Head*

1) Interfrontal setae: 0: absent or scattered across frons; 1: present in a distinct row. This character is usually regarded as a synapomorphy for the Sphaeroceridae. The derived state of this character is also found in members of the Milichiidae, and is approached in the Australian genus *Borboroides*, originally described as a sphaerocerid but now placed in the Heleomyzidae.
2) Ocellar bristle position: 0: posterior to median ocellus; 1: lateral or anterior to median ocellus.
3) Orientation of ocellar bristles: 0: proclinate; 1: reclinate.
4) Postocellar bristles: 0: absent or only weakly developed; 1: present, about as long as ocellar bristles.
5) Occipital setae: 0: covering lateral occiput, not in rows; 1: restricted to margin of occiput, in one or two rows.
6) Pseudotrachea: 0: 12; 1: fewer than 12; 2: more than 12. Almost all Archiborborinae have 12 pseudotrachea, although a few taxa have fewer or more. Although the exact number varies widely in other taxa, most Limosininae have fewer while most Copromyzinae have more.

*Thorax*

7) Sculpture of scutum: 0: not foveolate; 1: foveolate.
8) Dorsocentral bristles, female: 0: none; 1: one; 2: two; 3: three; 4: five. Three dorsocentral bristles is the ancestral state for the Sphaeroceridae. States 2 and 4 are only found in the outgroup. The anterior dorsocentral bristles are very short in males of many Archiborborinae and are difficult to code confidently.
9) Lateral bristles of mesoscutum: 0: normal size; 1: enlarged
10) Scutellum: 0: short, evenly curved; 1: long, subtriangular.
11) Anepisternum sculpture: 0: smooth; 1: with a gibbose triangle in posteroventral corner.
12) Katepisternal bristle: 0: absent; 1: present.
13) Metapleuron: 0: normal size; 1: enlarged

*Wing*

14) Wing: 0: normal to reduced, not rod-like; 1: reduced to a rod with apical bristle; 2: absent.
15) Vein M: 0: reaching costa; 1: ending in membrane before costa. Vein M is usually truncated in members of the subfamily Limosininae and is often used in keys to diagnose the subfamily.
16) Spots on vein CuA1: 0: absent; 1: present.
17) Colour of spots around crossvein: 0: light; 1: dark. For taxa that lack developed wings, this character and character 37 were coded as missing.
18) Patch of flattened setae on margin of calypter: 0: absent; 1: present.

*Legs*

19) Fore basotarsomere, male: 0: without spur; 1: with spur.
20) Mid tibia, row of anterodorsal bristles: 0: absent; 1: present. The number of bristles in this row varies within most species; this character was coded as present if at least one anterodorsal bristle is present.
21) Mid tibia, preapical posterodorsal bristles: 0: absent; 1: present.
22) Mid tibia, preapical anteroventral bristles: 0: absent; 1: one present; 2: two present.
23) Mid tibia, preapical posteroventral bristles: 0: absent; 1: present.
24) Hind tibia, anteroventral bristle: 0: absent; 1: present.
25) Hind tibia, anterodorsal bristles: 0: absent; 1: present.
26) Hind tibia, posterodorsal bristles: 0: absent; 1: present.
27) Hind tibia, ventroapical bristles: 0: absent; 1: one present; 2: two present; 3: three present.
28) Shape of hind basotarsomere: 0: thin, longer than second tarsomere; 1: swollen, shorter than second tarsomere. The derived state of this character is frequently used to diagnose members of the family Sphaeroceridae in keys.

*Abdomen*

29) Syntergite 1+2: 0: fully sclerotized; 1: with an anteromedial weakly sclerotized notch; 2: with only posterolateral corners and a thin connecting bar sclerotized.
30) Syntergite 1+2 sculpture: 0: not sculptured; 1: with a raised median ridge.
31) Fusion of syntergite 1+2 and tergite 3: 0: not fused; 1: fused.
32) Sternites 2–4, male: 0: heavily sclerotized; 1: weakly sclerotized.
33) Sternites 2–4, female: 0: heavily sclerotized; 1: weakly sclerotized.
34) Tergite 5: 0: heavily sclerotized; 1: weakly sclerotized
35) Lateral setae on tergites: 0: not strengthened; 1: strong, spine-like.

*Male genitalia*

36) Sternite 5 apodeme: 0: absent; 1: present
37) Sternite 5 apodeme keel: 0: keel absent; 1: keel present
38) Sternite 5 posterior margin: 0: round or slightly notched; 1: lateral corners produced, with tufts of long setae; 2: very broad, longest medially; 3: broad, with medial notch; 4: broad, with paired medial lobes; 5: lateral corners detached
39) Tergite 6: 0: present; 1: absent. This character is only present in *Tucma* among the Sphaeroceridae, although it is widespread among other Acalyptratae. Marshall [12] suggested this indicated a sister-group relationship between *Tucma* and the rest of the family.
40) Epandrium: 0: without a cleft above surstylus; 1: with a cleft above surstylus.
41) Hypandrium: 0: without ventral tab; 1: with ventral tab. The ventral tab is a transparent extension of the hypandrium near the apex of the lateral arm.
42) Cerci: 0: not fused below anus; 1: fused below anus.
43) Cerci: 0: immediately below anus; 1: separated from anus by an extension of the epandrium.
44) Surstylus: 0: ventral margin complete; 1: ventral margin with a deep notch.
45) Basiphallus: 0: normal; 1: swollen.
46) Distiphallus, dorsal tube: 0: absent; 1: present. The dorsal tube is a unique, tube-like structure in many Archiborborinae. It often bears a number of small tooth-like spicules or pseudotrichia.
47) Distiphallus, dorsobasal fork: 0: absent; 1: present. The dorsobasal fork is a flattened, fork-shaped sclerite near the base of the distiphallus found in some Archiborborinae.

*Female genitalia*

48) Ovipositor sclerites: 0: sclerites as long as wide or longer; 1: sclerites much wider than long
49) Ovipositor: 0: with posterior weakly sclerotized strips; 1: without posterior strips
50) Tergites 6 and 7, female: 0: not sclerotized medially; 1: evenly sclerotized
51) Epiproct: 0: without setae; 1: with setae
52) Cercus, female: 0: shiny; 1: with microtomentum
53) Spermathecae: 0: 2 present; 1: 3 present. The number of spermathecae can be a plastic character, even varying within some genera of Sphaeroceridae, but may be a valuable character for higher level phylogenetics in some cases. This character could not be coded for *Pycnopota*, as the spermathecae could not be located in the single teneral female available for dissection.

### Molecular characters

Portions of five genes were sequenced: mitochondrial 12S rDNA (portion of 5′ end, 358 bp), cytochrome c oxidase subunit I (COI; Folmer or barcode region of 5′ end, 655 bp), and cytochrome B (CytB; 3′ end, 712 bp), and nuclear alanyl-tRNA synthetase (AATS; portion of 5′ end, 401 bp) and 28S rDNA (5′ end encompassing expansion segments D1 and D2, 662 bp). The data set thus includes both mitochondrial and nuclear genes, and both ribosomal and protein-coding genes. Genes were selected based on the availability of primers, expected rate of evolution, and prior successful use in phylogenetic analyses of Diptera. A few additional species were sequenced for COI only by the Canadian Center for DNA Barcoding following the methodology of Smith et al. [13] (all COI sequences generated for the project are available with Genbank accession numbers JX260352–JK260397). All sequences are available on Genbank, and sequences and supporting trace files are available from the BOLD workbench [14]. Specimen voucher numbers, accession numbers and BOLD sample numbers are listed in Table S1. Sequences for *Rachispoda* sp. (from [8]) were downloaded from Genbank.

### Extraction

Most specimens used in the analysis were stored in alcohol, except the representatives of *Archiborborus orbitalis* Duda and undescribed species “siberia”, and “echinus”, for which dried specimens or legs thereof were used. Extractions were performed with the DNeasy® Blood & Tissue Kit (Qiagen Inc., Santa Clara, CA, USA), using entire specimens when possible. Procedure followed the recommended spin-column protocol, with the specimen left in the lysis solution for approximately 16–20 hours, and with the final centrifugation step modified by eluting with 200 µL of pH 8.9 water instead of buffer. This solution was then dried in a DNA110 SpeedVac (ThermoSavant) before resuspension of the DNA in 50 µL of buffer. Extracted specimens were stored in ethanol until they could be dried in a critical point drier and mounted for morphological study. All specimens are deposited in the University of Guelph Insect Collection except the specimens of undescribed species “marensis” and “crocidosternum”, which are deposited in the collection of the Museu de Zoologia, Universidade de Sao Paulo.

### Amplification and purification

DNA amplification was performed in 25 µL volumes; as concentration of extractions varied considerably, up to 10 µL of template was used with the volume of H_2_O reduced accordingly. Reactions using Taq polymerase (Promega Corp., Madison, WI, USA) included 0.5 µL of polymerase, 2.5 µL of 10× PCR buffer, 2.5 µL of MgCl_2_ (25 mM), 0.5 µL each of forward and reverse primers (10 µM), 0.5 µL of 10 µM dNTPs, 2–10 µL of template, and 9–16 µL of ddH_2_O. Reactions using ExTaq polymerase (Takara Bio USA, Madison, WI, USA) included 0.125 µL of polymerase, 2.5 µL of 10× ExTaq buffer, 0.625 µL of MgCl_2_ (25 mM), 1.0 µL each of forward and reverse primers (10 µM), 2.0 µL of dNTPs (10 mM), 0.5–10 µL of template, and 7.7–17.3 µL of ddH_2_O. PCR programs, specific to each primer program, were run in an Eppendorf epGradient S Mastercycler (Eppendorf AG, Hamburg, Germany). Primers and polymerase used are described in Table 1. All products were visualized on 1% agarose electrophoresis gels with 2.7% ethidium bromide (10 mg/mL) using UV transillumination.

**Table 1:** Primers used for amplification and sequencing. References indicate the first publication of the primer.

Gene (direction)	Primer	Sequence
12S (f)	12Sbi [15]	AAGAGCGACGGGCGATGTGT
12S (r)	12Sai [15]	AAACTAGGATTAGATACCCTATTAT
12S (r)	12S-Dipt-14525R [16]	CGGTATTTTAKTCTDTYCAGAGG
COI (f)	LCO1490 [17]	GGTCAACAAATCATAAAGATATTGG
COI (r)	HCO2198 [17]	TAAACTTCAGGGTGACCAAAAAATCA
COI (r)	COI-Dipt-2183F [16]	CAACAYTTATTTTGATTTTTTGG
CytB (f)	CB-J-10933 [15]	TATGTTTTACCTTGAGGACAAATATC
CytB (r)	TS1-N-11683 [15]	AAATTCTATCTTATGTTTTCAAAAC
AATS (f)	1F40 [10]	GNATGAAYCARTTYAARCCNAT
AATS (f)	AATS-Dipt-562F [16]	CGNGCHGGHGGHAARCAYAAYGA
AATS (f)	AATS-Dipt-611F [16]	TAYCAYCAYACNTTYTTYGARATG
AATS (r)	1R244 [10]	CATNCCRCARTCNATRTGYTT
AATS (r)	AATS-Dipt-955R [16]	CGATTRWAYTGWATRAANACHARRTTCC
28S (f)	28S-Dipt-3385F [16]	GGATTTTCTTAGTAGCGGCG
28S (r)	28B [18]	CCCGTCTTGAAACACGGACC

PCR products were purified either using ExoSAP-IT® (USB Corp., Cleveland, OH, USA) following the manufacturer's instructions, with a QIAquick® Gel Extraction kit (Qiagen Inc., Santa Clara, CA, USA) following the manufacturer's instructions, or using CloneWell 0.8% SYBR Safe™ precast gels in the E-Gel® system (Invitrogen™, Carlsbad, CA, USA) [19].

### Sequencing

Purified products were prepared for sequencing using an ABI BigDye® Terminator v3.1 Cycle Sequencing kit (PE Applied Biosystems, Foster City, CA, USA). DNA sequencing was performed at the Agriculture & Agri-Food Canada Eastern Cereal and Oilseed Research Centre Core Sequencing Facility (Ottawa, ON, Canada) on an ABI 3130xl Genetic Analyzer (PE Applied Biosystems, Foster City, CA, USA) using the ABI ethanol/EDTA/sodium acetate protocol. Sequence chromatograms were viewed and contigs assembled in BioEdit [20].

### Alignment

Alignment for 12S, COI, CytB, and AATS was performed using the Clustal algorithm implemented in BioEdit. Alignment was straightforward for these genes, with the only indel consisting of an amino acid insertion in AATS for *Apteromyia*. Alignment of the expansion segments of 28S is difficult with standard alignment algorithms, and so an alternative procedure was followed for this gene. Initial alignment was performed manually based on the published secondary structure for *Drosophila melanogaster* [21]; the expansion segments were then identified and aligned separately with the program LocARNA [22], which performs multiple alignment based on predicted folding properties of RNA sequences. A single loop region in expansion segment D2, corresponding to positions 454 to 460 in the aligned sequence, was highly variable in length and composition between taxa. It could not be aligned with confidence and was excluded from analysis. The aligned dataset is available in Nexus S2.

### Data analysis

Analyses were based on morphological data for 128 taxa (116 Archiborborinae and 12 outgroup taxa), molecular data for 28 exemplar taxa, and combined data for both the 28 exemplar taxa and all 128 taxa (the latter including additional COI sequences from species not included in the molecular-only analysis). All data sets were analysed using both parsimony and Bayesian methods.

Parsimony analyses were conducted in TNT (Willi Hennig Society edition [23]). All characters were treated as unordered and reversible. Bremer indices were calculated for the strict consensus of each analysis. Gaps in the molecular data were treated as missing characters. The search strategy for trees was carried out in two steps; first a New Technology search incorporating sectoral search and tree fusing to find the minimum length 10 times, followed by a traditional (heuristic) search to use TBR swapping on the trees found in the previous step. Character transformation was analyzed in PAUP* 4.b10 [24] with ACCTRAN optimization.

Partitioning strategies for the molecular components of the Bayesian analysis were analysed using Phycas [25]. Phycas implements a stepping stone method for accurate calculation of the marginal likelihoods of different models, allowing comparison of different partitioning strategies [26]. Four different partitioning strategies were compared: no partitioning, partitioning by gene (5 partitions), partitioning by codon position for all three protein-coding genes (5 partitions), and partitioning by gene and codon position (11 partitions). The initial reference tree was generated in Phycas using partitioning by gene and allowing polytomies, and used for the marginal likelihood calculations (β = 11, with 1000 cycles per β value). Marginal likelihoods were then compared using Bayes factors [27].

Bayesian analyses were conducted using MrBayes versions 3.1.2 and 3.2.1 [28]–[30]. Some MrBayes runs were completed through the CIPRES Science Gateway implemented on the Trestles TeraGrid cluster [31]. All parameters except topology and branch lengths were unlinked between partitions. Analyses consisted of 2 runs with 4–6 chains each, and were run for 10–60 million generations, depending on how rapidly convergence was reached. Temperature was set to 0.08 to improve mixing and convergence. Convergence was assessed using the *cumulative*, *slide* and *compare* options in AWTY [32] to assess split frequency within and between runs, respectively. Trace plots of model parameters were also examined using Tracer [33] to assess mixing and the adequacy of priors.

Rather than determining the nucleotide substitution model for each partition *a priori*, we used the *nst = mixed* setting implemented in MrBayes 3.2. This setting allows the MCMC analysis to sample across the GTR model space [34]. For all partitions, among-site rate variation was accommodated using gamma-distributed rates (+Γ). Although substitution models incorporating a parameter for a proportion of invariable sites (+I) are often used in phylogenetic analyses, gamma-distributed rates can accommodate a wide range of substitution rates, and combining +Γ and +I parameters in partitioned data sets can result in unreasonable parameter values in the model [26]. The morphological data partition was analysed under the Mk+Γ model [35]; transition rate asymmetry was accomodated by setting the hyperprior for the symmetric Dirichlet distribution to *exponential(1.0)*. As only informative characters were included in the morphological dataset, the coding option was set to *inf*.

For analyses with the full taxon set, the consensus trees were poorly resolved. To test whether a few unstable taxa were obscuring the phylogenetic signal, we used the RogueNaRok web service [36]. For parsimony analyses, we optimized the number of bipartitions in the strict consensus trees; for Bayesian analyses, we optimized the support in the majority-rule consensus. In both cases, the default RogueNaRok algorithm was used. Taxa identified as potential wildcards were pruned from trees if their removal improved the resolution or support of the backbone of the tree.

## Results

### Morphological analysis

**Figure 3:**
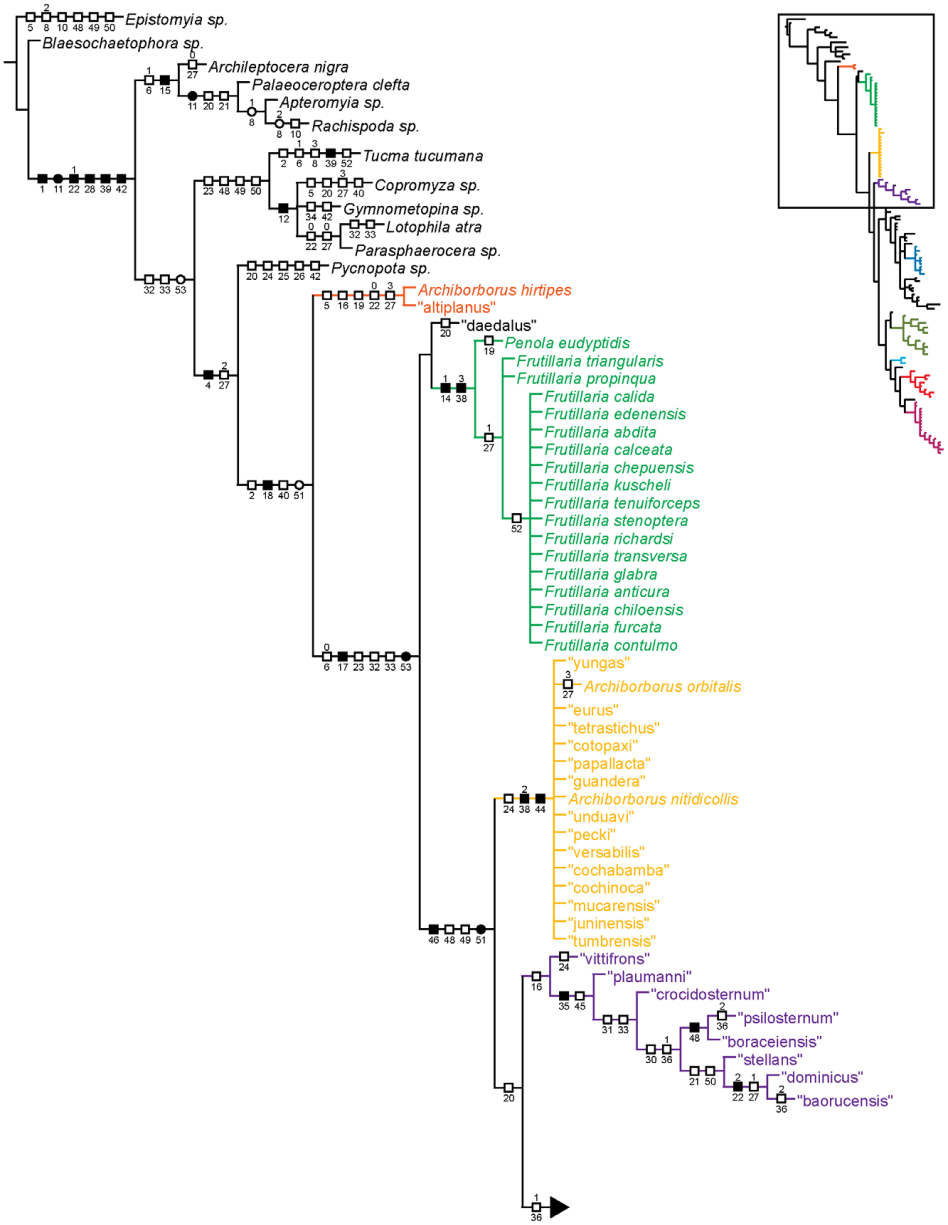
Parsimony analysis of morphological dataset, representative tree. Character changes were optimized using ACCTRAN transformation. Boxes indicate unambiguous character changes on a branch, circle represent ambiguous changes; filled boxes or circles are unreversed changes. Numbers below branches indicate character number, numbers above branches indicate character state for multi-state characters.

**Figure 4:**
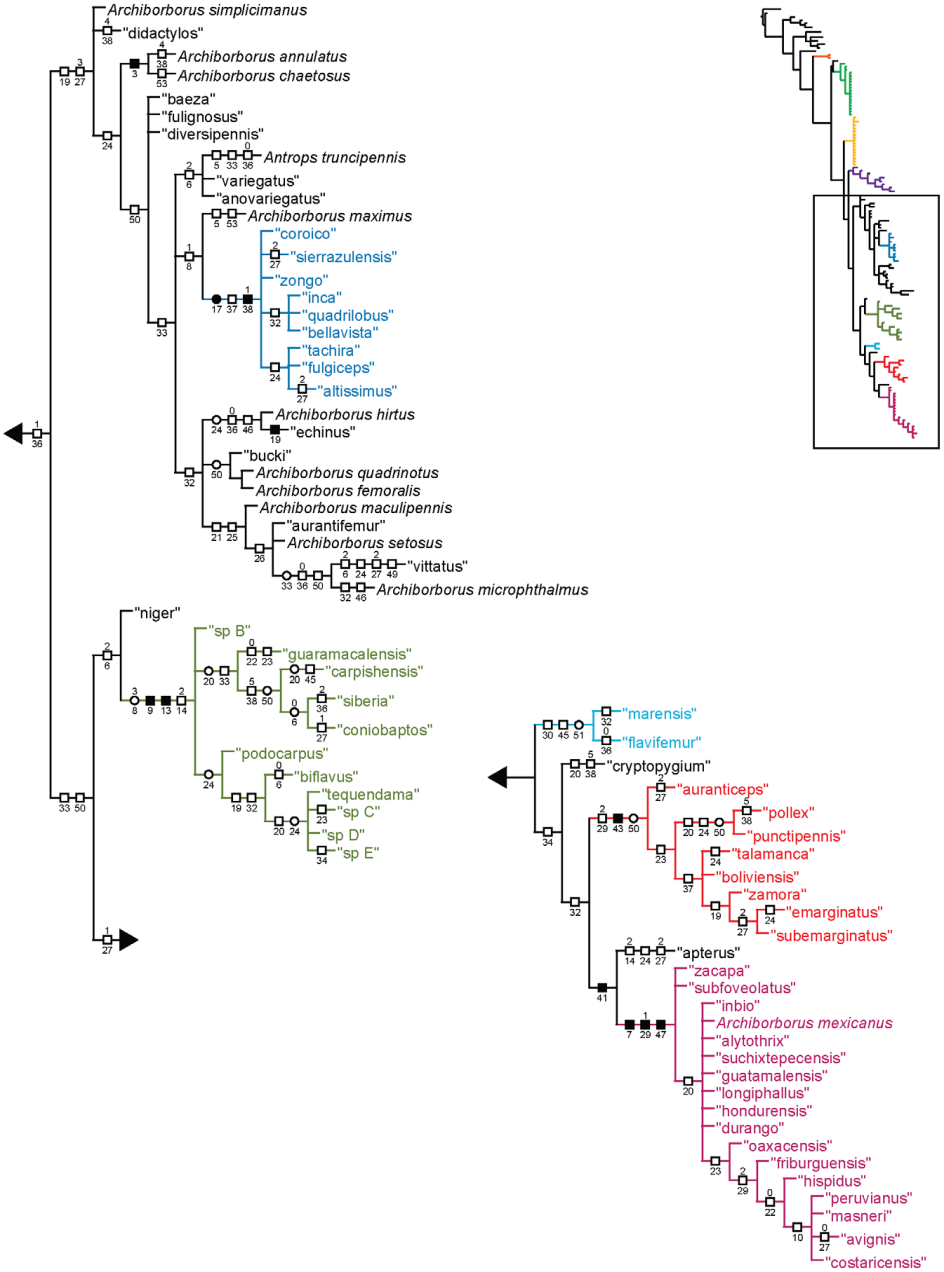
Parsimony analysis of morphological dataset, representative tree, continued. Character changes were optimized using ACCTRAN transformation. Boxes indicate unambiguous character changes on a branch, circle represent ambiguous changes; filled boxes or circles are unreversed changes. Numbers below branches indicate character number, numbers above branches indicate character state for multi-state characters.

Parsimony analysis of the morphological dataset yielded a large number of trees (length 210, CI: 0.319, RI: 0.850). A set of 10,000 trees was retained for further analysis; saving additional trees did not affect the consensus (Figures 1, 2). Archiborborinae were recovered as monophyletic, supported by three unambiguous character changes (mapped in Figures 3, 4) — the cleft epandrium (character 40, shared with *Copromyza* sp. as well as other Copromyzinae not included in the matrix), ocellar bristles anterior of anterior ocellar triangle (character 2, shared with *Tucma tucumana*), and the dense patch of setae on the alula (character 18, a unique synapomorphy). Within the Archiborborinae, we have given informal group names (based on distinctive characters or representative species) to several of the clades recovered as monophyletic to simplify further discussion. Of these groups, the *spotted wing* group, consisting of *Archiborborus hirtipes* and an undescribed sister species (“altiplanus”), was recovered as sister to all other archiborborines. The clade consisting of *Penola* and *Frutillaria* formed a basal polytomy with the undescribed species “daedalus” and the remaining archiborborines. The remaining species largely formed an unresolved bush, with some clustered into clades within the bush. Bremer indices were low, with most nodes collapsing in trees 1 step longer. Three additional clades were recovered in the strict consensus after pruning the taxa “apterus” and “biflavus”: one comprising most of the taxa in the bush except the *colourful legs* and *orbitalis* groups, one comprising a group of wingless species (*apterous* group), and one including the *emarginatus* and *mexicanus* groups.

**Figure 5:**
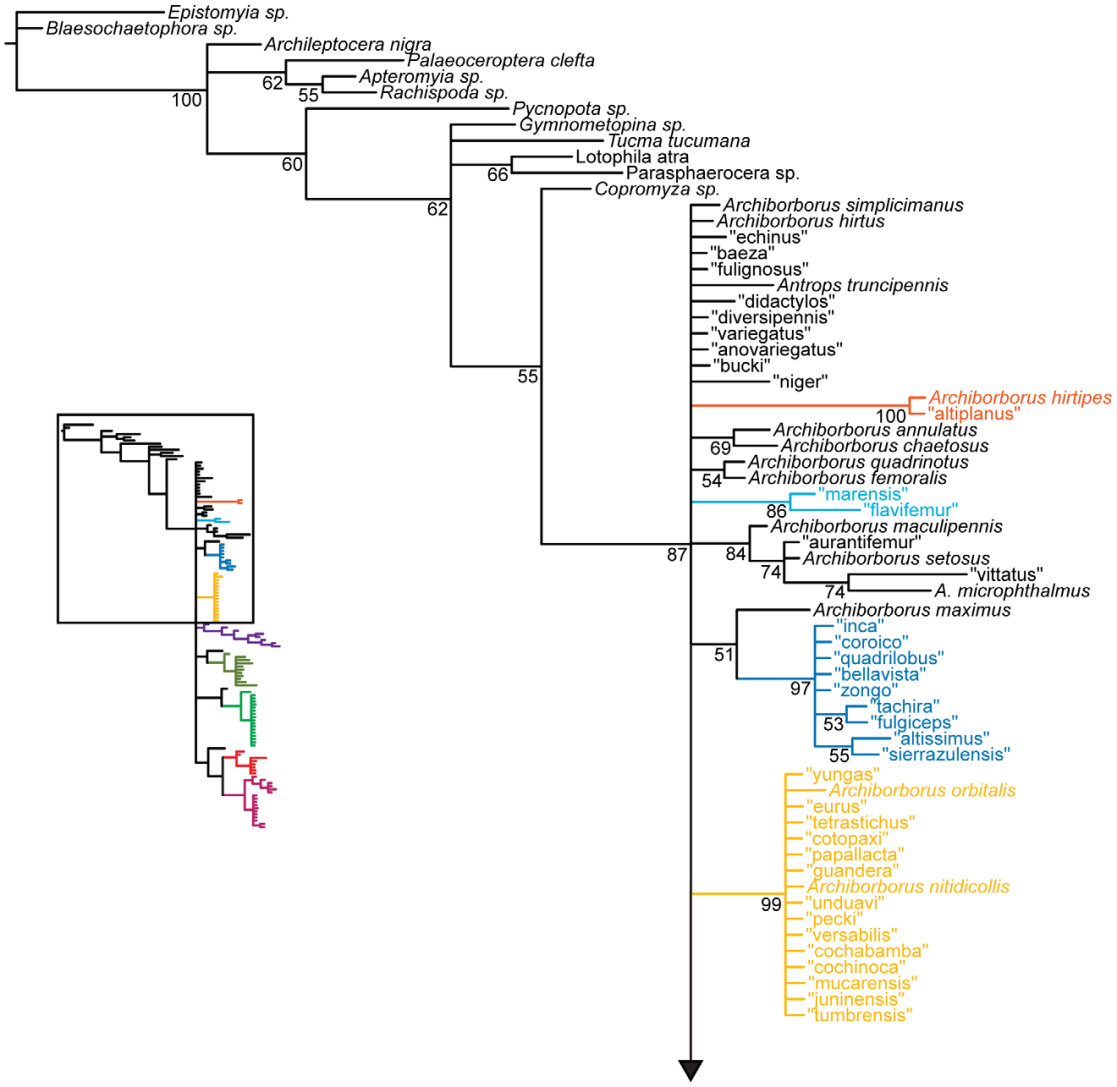
Bayesian analysis of morphological dataset. Numbers beneath nodes are posterior probabilities.

**Figure 6:**
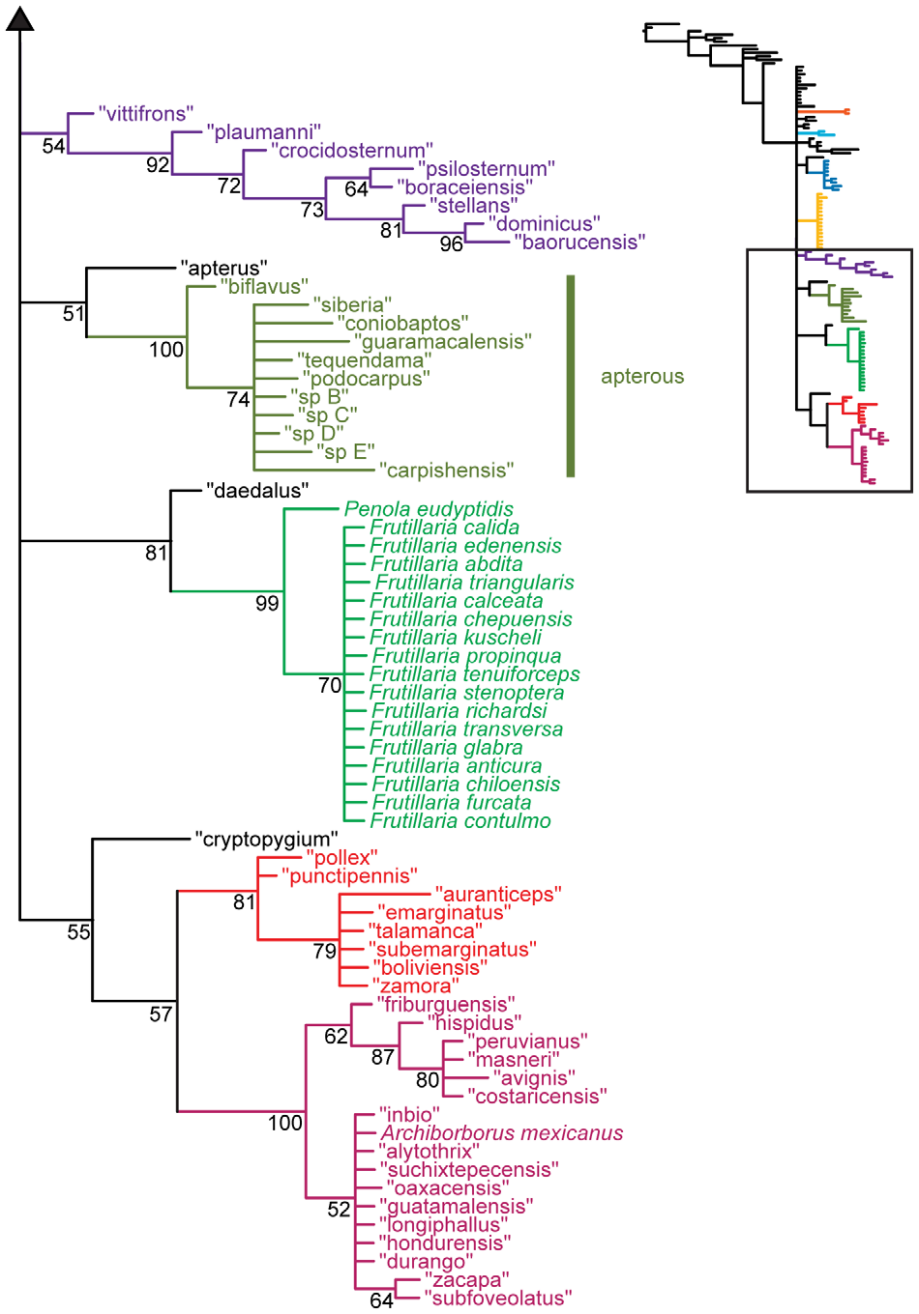
Bayesian analysis of morphological dataset, continued. Numbers beneath nodes are posterior probabilities.

Bayesian analysis of the morphological dataset yielded a monophyletic Archiborborinae (pp = 0.87), but with most species groups branching from a single polytomy (Figures 5, 6). Both the spotted wing group and *Penola* + *Frutillaria* were recovered in this unresolved bush. The species groups recovered in the parsimony analysis were also recovered as monophyletic, many with high posterior probabilities (eg. *quadrilobus*, 0.97; *orbitalis*, 0.99; *mexicanus*, 1.0). A few taxa were found to increase support for various subclades when pruned, but none improved resolution within the large bush and so we present results with all taxa included.

### Molecular analysis

Sequence data were obtained for 25 (COI) to 27 (12S) taxa for each gene fragment, and data for one additional taxon were obtained from Genbank. Between 10.3% (12S) and 35.0% (CytB) of the bases for each gene were parsimony informative, while G+C content ranged from 23.5% (12S) to 46.8% (AATS) (Table 2). The partitioning strategy test showed that partitioning by gene and codon was better than the other models by a considerable margin (Table 3), and so this strategy was used for all analyses.

**Table 2:** Characteristics of sequence data.

Gene	No. of taxa	PI bp/total bp (%)	G+C content
12S	28	37/358 (10.3)	23.5%
COI	26	192/655 (29.3)	33.5%
CytB	27	249/712 (35.0)	27.4%
AATS	27	120/401 (30.0)	46.8%
28S	25	98/662 (14.8)	32.6%

**Table 3:** Results of Bayesian partition analysis.

Partition strategy	No. of partitions	ln likelihood	2ln(BF)
no partitions	1	−19091.73	
by gene	5	−17764.93	2653.6
by codon	5	−17298.77	932.32
by gene and codon	11	−16408.17	1781.2

For each model, marginal ln likelihood was calculated using Phycas. The comparison statistic 2ln(BF) was calculated following [23]. Each value is presented as a comparison with the next-best model; values over 10 are considered strong support for a given model.

**Figure 7:**
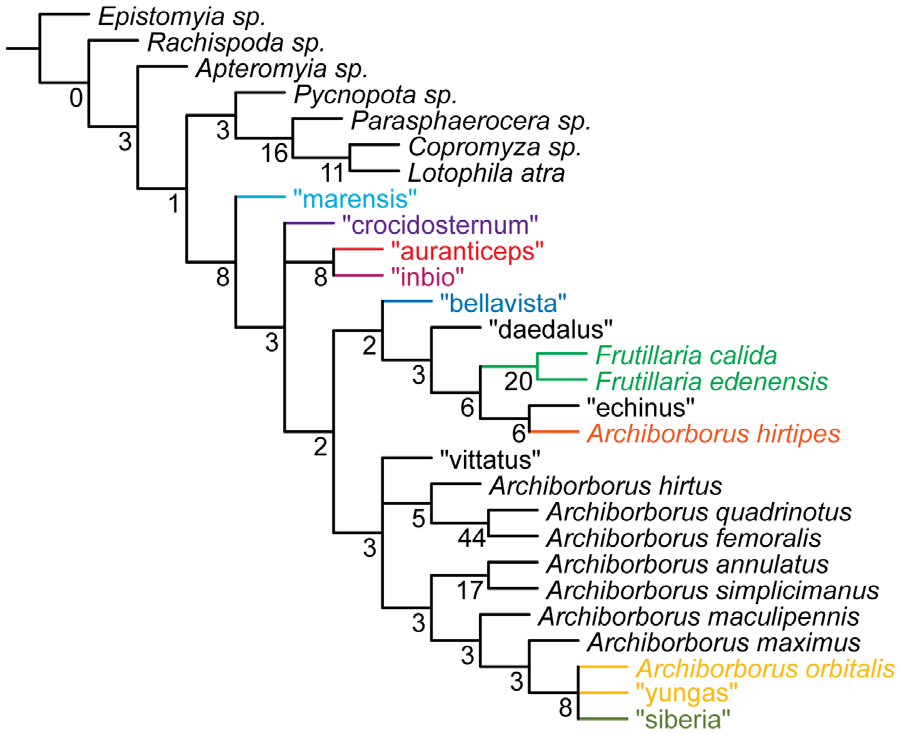
Parsimony analysis of molecular dataset Strict consensus of 3 trees, length 3569. Numbers beneath nodes are Bremer support indices.

**Figure 8:**
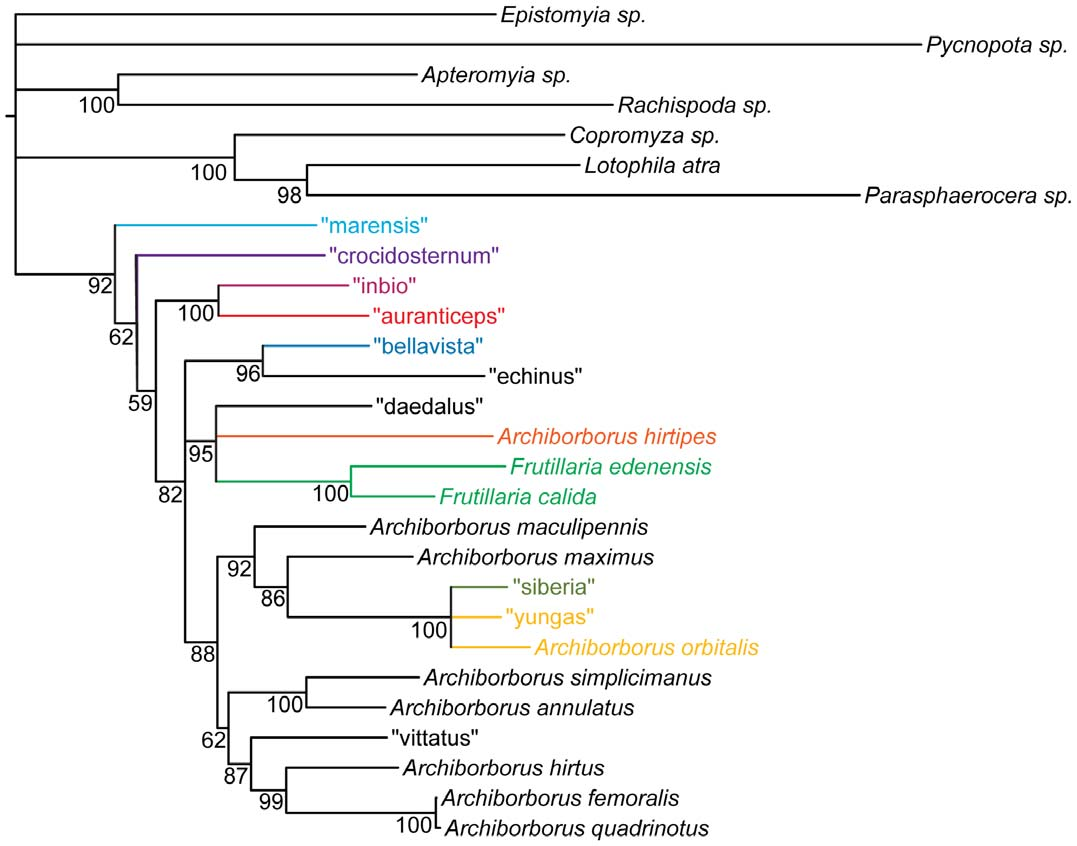
Bayesian analysis of molecular dataset. Numbers beneath nodes are posterior probabilities.

Parsimony analyses produced a well-resolved consensus (Figure 7; 3 trees, length 3569, CI: 0.368, RI: 0.345). The analysis placed the representatives of the *colourful legs*, *yellow forelegs*, *mexicanus*, and *emarginatus* groups as sister to the remaining archiborborines, with the *mexicanus* and *emarginatus* representatives sister to each other. Bremer indices for the backbone were low. The Bayesian analysis was also well resolved, with moderate to strong support along the backbone (Figure 8). The same four exemplar species were recovered as sister to the remaining archiborborines. Positions of the remaining ingroup taxa were also similar, although with some rearrangements.

### Combined analysis

**Figure 9:**
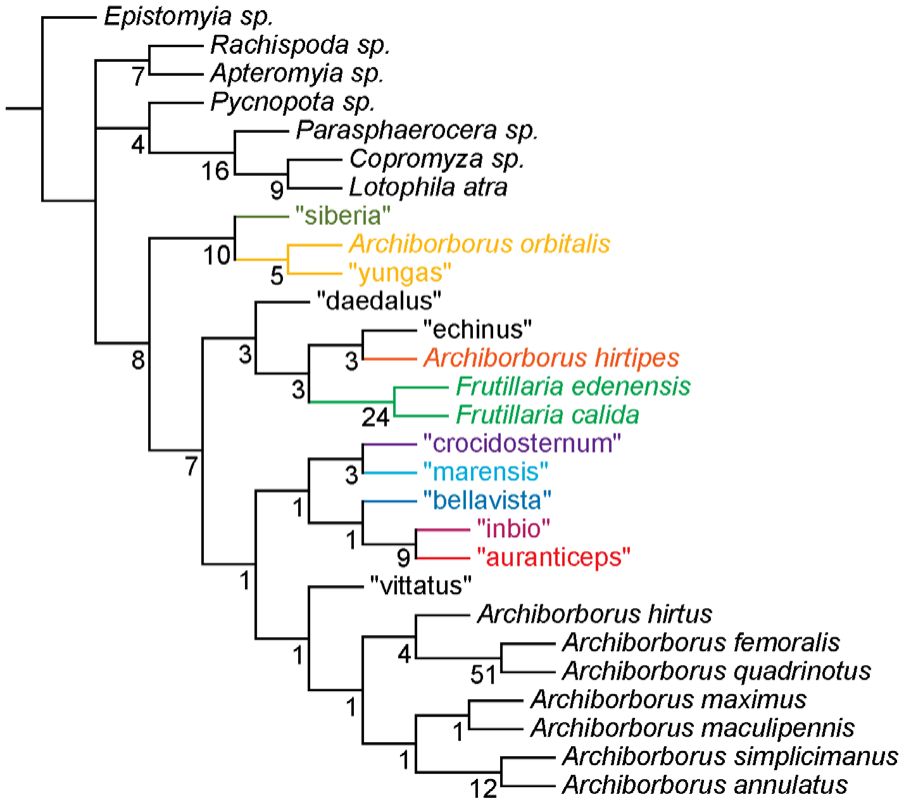
Parsimony analysis of combined dataset, 28 taxa. Strict consensus of 2 trees, length 3734. Numbers beneath nodes are Bremer support indices.

**Figure 10:**
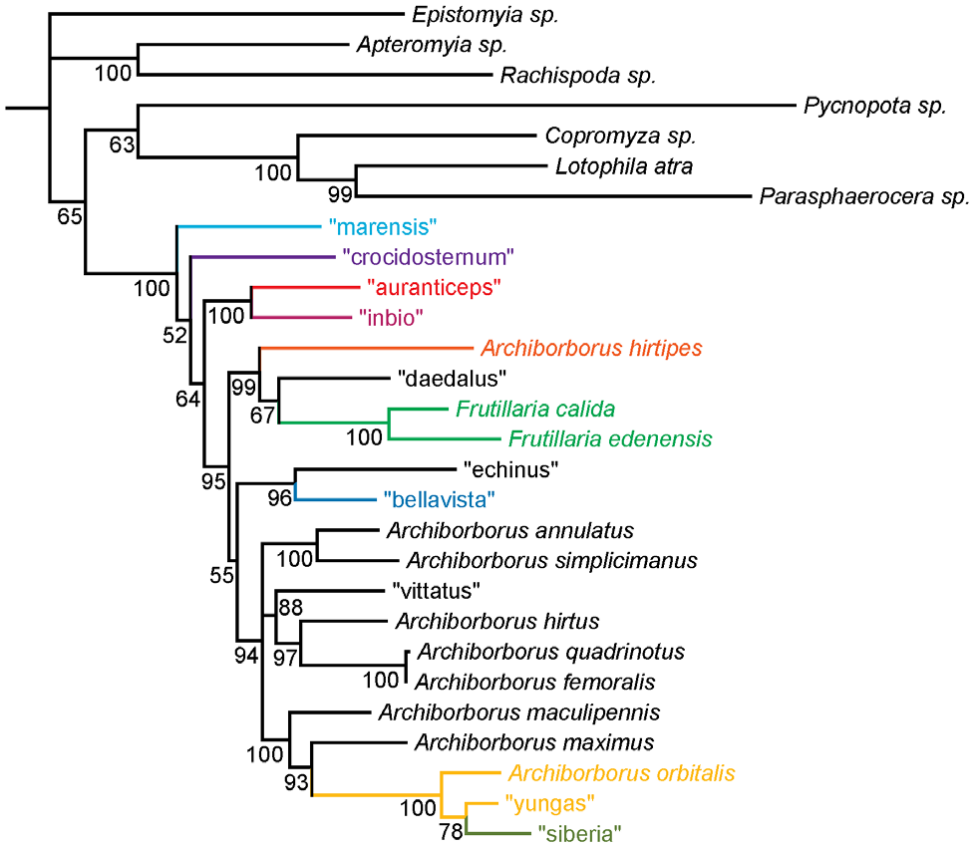
Bayesian analysis of combined dataset, 28 taxa. Numbers beneath nodes are posterior probabilities.

Parsimony analysis of the 28 taxon combined data set yielded two parsimonious trees (length 3734, CI: 0.372, RI: 0.356). The strict consensus (Figure 9) had a very different topology from those recovered in the molecular analysis, with the representatives of the *apterous* and *orbitalis* groups recovered as sister to the remaining archiborborines. The taxa that had been recovered as the basal lineages in the molecular analysis were found to form a weakly supported clade, along with “bellavista”. The Bayesian analysis, however, produced a topology very similar to the molecular analysis (Figure 10). Posterior probabilities were similar, with only slightly higher or lower support for most clades.

**Figure 11:**
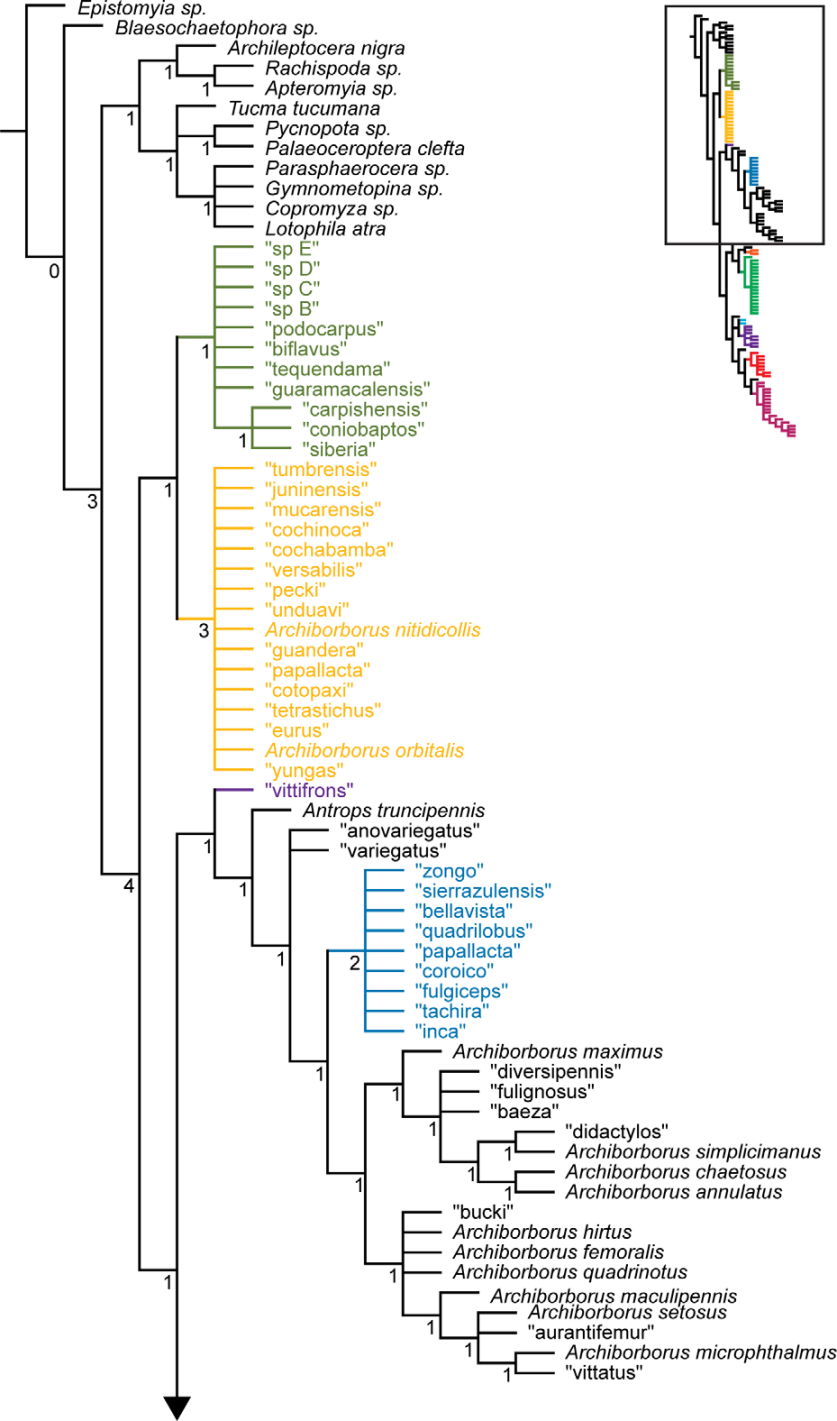
Parsimony analysis of combined dataset, 128 taxa. Strict consensus of 10000 trees, length 4041. Numbers beneath nodes are Bremer support indices.

**Figure 12:**
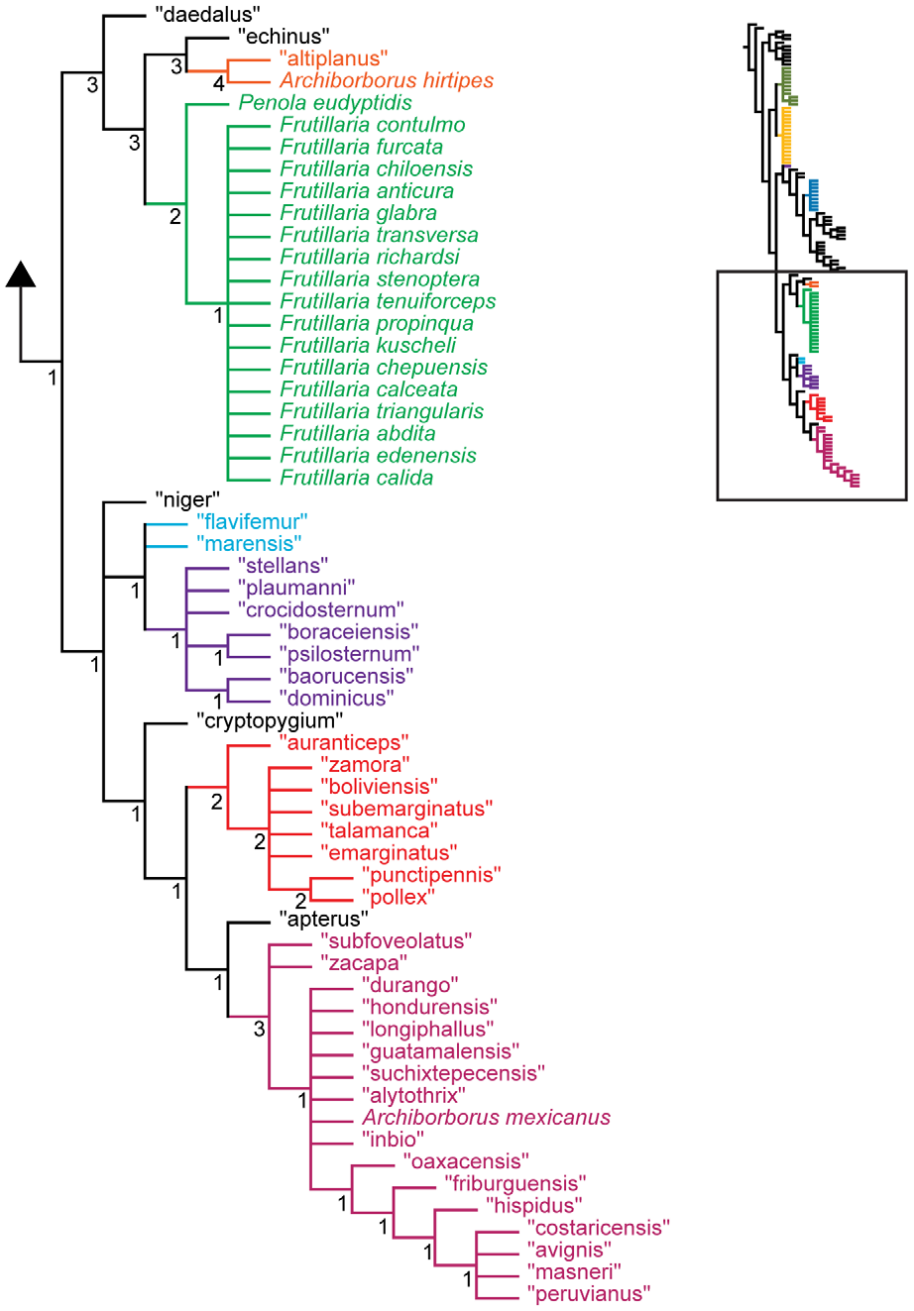
Parsimony analysis of combined dataset, 128 taxa, continued. Strict consensus of 10000 trees, length 4041. Numbers beneath nodes are Bremer support indices.

**Figure 13:**
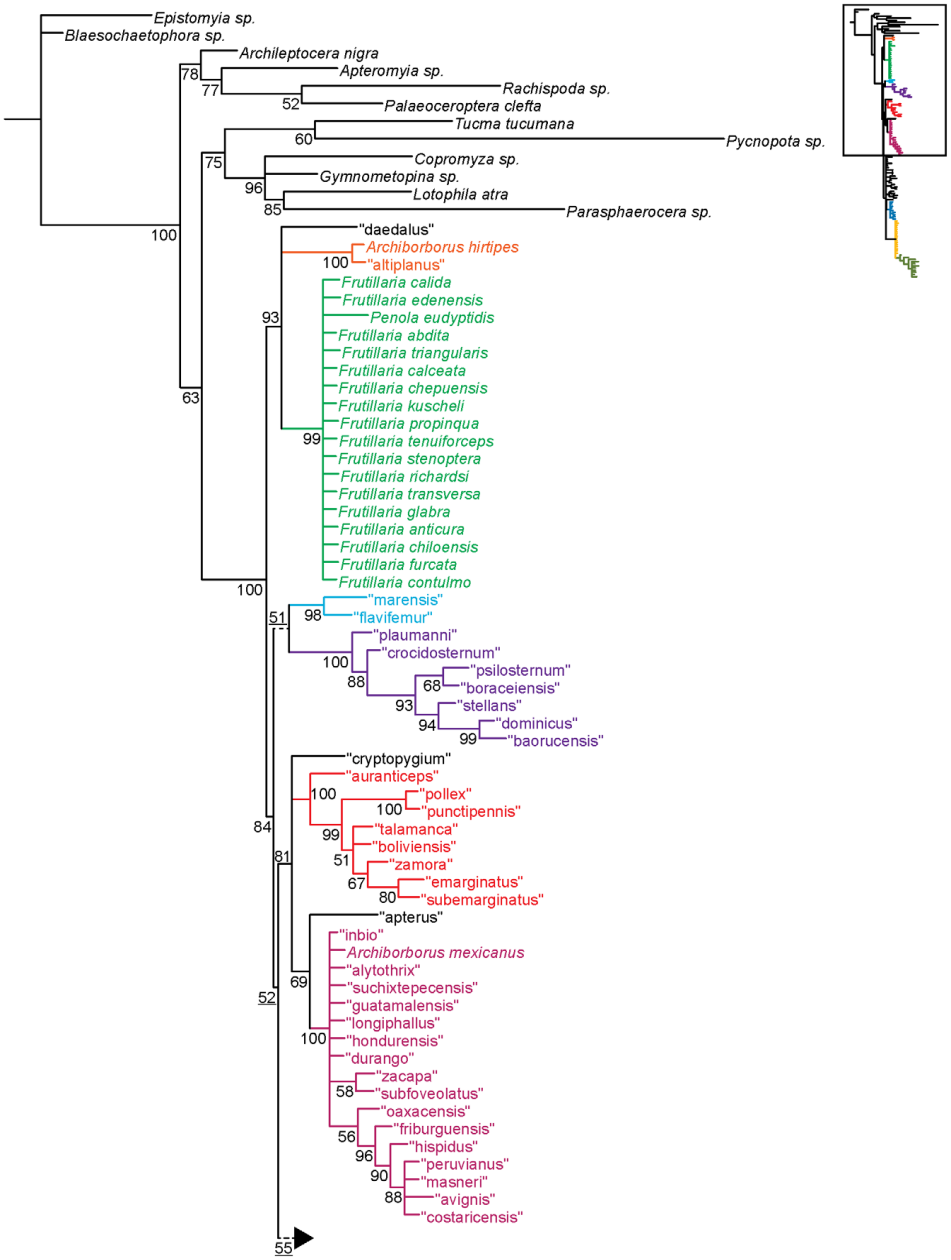
Bayesian analysis of combined dataset, 128 taxa. Numbers beneath nodes are posterior probabilities. Dashed branches with underlined support values indicate bipartitions only recovered about 0.5 posterior probability after pruning the taxa “echinus” and “vittifrons”.

**Figure 14:**
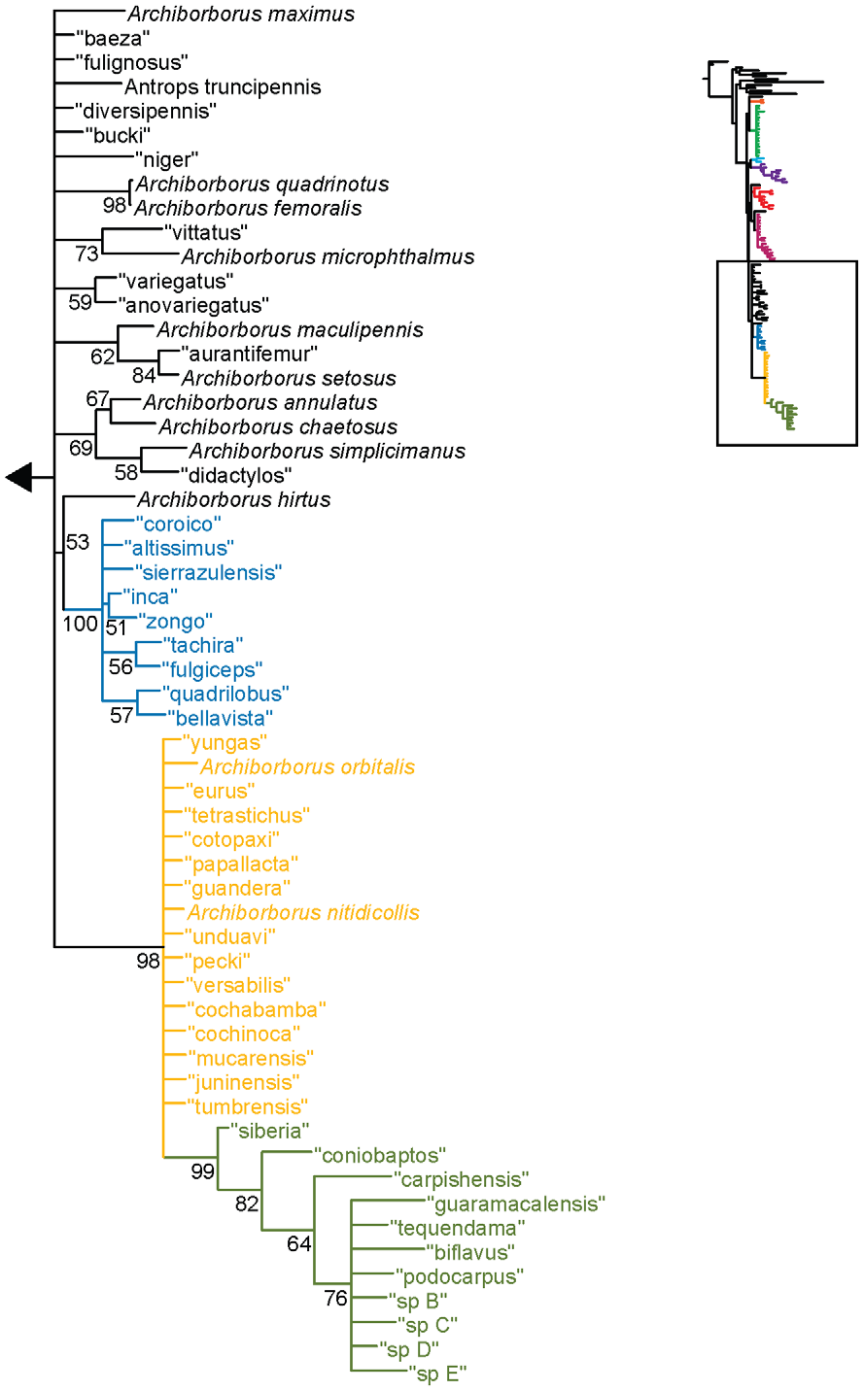
Bayesian analysis of combined dataset, 128 taxa, continued. Numbers beneath nodes are posterior probabilities. Dashed branches with underlined support values indicate bipartitions only recovered about 0.5 posterior probability after pruning the taxa “echinus” and “vittifrons”.

The parsimony analysis of the 128 taxon data set produced a very large number of equally parsimonious trees (length 4041, CI: 0.349, RI: 0.453). As with the morphological analysis, 10,000 trees were retained for further analysis. The strict consensus tree (Figures 11, 12) was fairly well resolved. The *apterous* and *orbitalis* groups were placed as sister to each other, and together as sister to the remaining archiborborines. Many of the species placed in the large unresolved bush in the morphological analysis were recovered in a single clade, also including the *quadrilobus* group. However, Bremer support indices were low. The Bayesian analysis (Figures 13, 14) was not as well resolved; as with the morphological dataset, most species and groups were recovered in a single bush. Unlike the parsimony analysis, a clade including the spotted wing group, *Penola* and *Frutillaria*, and “daedalus” was recovered as sister to the remaining Archiborborinae; the clade exclusive of those taxa was supported with moderate posterior probabilities (pp = 0.84). Pruning two taxa, “vittifrons” and “echinus”, improved support somewhat and added some resolution to the backbone. Specifically, two clades comprising the *yellow forelegs* and *colourful legs* groups and the *emarginatus* and *mexicanus* groups respectively were recovered as subtending a clade containing the remaining taxa and groups. However, support for this arrangement was still very low, with posterior probabilities just over 0.5.

## Discussion

### Monophyly and position of the Archiborborinae

The Archiborborinae were supported as monophyletic in all analyses. There are several apparent morphological synapomorphies for the subfamily (Figures 3. 4). All fully winged species have a dense patch of flattened setae on the calypter (character 18); unlike other members of the family. This is the only unreversed unique synapomorphy for the subfamily, although it could not be coded for apterous species. When present, the ocellar bristles are inserted at or anterior to the level of the median ocellus (character 2); this character is shared only with *Tucma* in the Sphaeroceridae. The postocellar bristles are also greatly enlarged in all species where they are present (character 4); this character also occurs in *Pycnopota* and some Limosininae, but not in the Copromyzinae. The epandrial cleft (character 40) was also recovered as a synapomorphy in the morphological analysis, shared only with *Copromyza*; however, this character occurs more widely in unsampled Copromyzinae and a possibly homologous state occurs in the Sphaerocerinae.

The positions of the Archiborborinae and the Copromyzinae in all analyses contradicted their previous treatment as a single subfamily. The Copromyzinae and Sphaerocerinae consistently formed a monophyletic clade to the exclusion of the Archiborborinae. Synapomorphies for the Sphaerocerinae-Copromyzinae clade include the loss of the katepisternal bristle (character 12) and probably the presence of only two spermathecae (character 53), although the latter is also widespread elsewhere in the Sphaeroceridae and is also found in the *spotted wing* group of Archiborborinae. In the molecular and combined analyses, the exemplars of *Lotophila* and *Parasphaerocera* formed a well-supported clade within this group, sister to the exemplar of *Copromyza*. While the sphaerocerines clearly form a monophyletic group within the Sphaerocerinae-Copromyzinae clade, there are no clear morphological synapomorphies for the Copromyzinae s.s. and it is probable that the subfamily is not monophyletic. Further phylogenetic analysis with more extensive taxon sampling in this clade is needed.

The position of the enigmatic taxon *Pycnopota* varied between analyses. None of the positions were well supported. Very little is known of this genus, which includes a single described species from Bolivia and undescribed species from Brazil (specimens in United States National Museum of Natural History, Smithsonian Institution, Washington and Museu de Zoologia, Universidade de Sao Paulo) and Costa Rica (specimens in University of Guelph Insect Collection). Roháček et al. [2] considered the genus a possible member of the Heleomyzidae, but other than the spinose costa its morphology is that of a sphaerocerid, and the phylogenetic results corroborate this. Final resolution of its position will require broader taxon sampling of non-archiborborine Sphaeroceridae and Heleomyzidae, but it is clearly distinctive and may represent a new monotypic subfamily.

The relationship between the Archiborborinae and the remaining Sphaeroceridae was moderately well resolved from the data. In most analyses it was placed in a clade with *Pycnopota* and the Sphaerocerinae+Copromyzinae clade, although this clade was only well supported in a few analyses. This grouping (excluding *Pycnopota*) was suggested by Marshall [37] as monophyletic based on the presence of the epandrial cleft. This character is present in all Archiborborinae and many Copromyzinae (apparently reversed in a clade of four genera, including *Lotophila* [38]), while the Sphaerocerinae have a possibly homologous state with the epandrium completely divided above the anus. However, final resolution of the position of the Archiborborinae and relationships of the subfamilies may require more extensive taxon sampling, particularly including molecular data for *Tucma* and problematic genera such as *Palaeoceroptera* Duda.

### Phylogenetic relationships of the Archiborborinae

Analyses produced mixed results within the Archiborborinae. The morphological and combined analyses supported fairly consistent species groups within the subfamily. However, the relationships between the species groups were inconsistent and generally poorly supported, and a number of species were not placed in species groups in some analyses. Basal nodes in particular were poorly resolved in all analyses, with short branch lengths; this may indicate that the subfamily underwent rapid divergence early in its evolutionary history.

The existing generic classification of the Archiborborinae [2] places all winged species in *Archiborborus*. Despite the conflicting results of different analyses, this classification is clearly untenable. Winged and brachypterous or apterous species occur throughout the subfamily. The all-taxa combined parsimony analysis suggests wing loss occurred seven times, while the all-taxa combined Bayesian analysis suggests at least five (and possibly seven) occurrences. Wing loss is quite common among the Sphaeroceridae; although no complete phylogeny for the family is available, flightless species are currently known in 30 genera in the family (unpublished data), and in at least some of those genera there have probably been multiple instances of wing loss (eg. *Aptilotus* Mik [39]–[40]). Clearly this character is not reliable as a primary basis for a cladistic classification. All analyses show that the type species of *Antrops*, *Penola*, and *Frutillaria* are within clades including winged species, rendering such a broad concept of *Archiborborus* paraphyletic. *Frutillaria* is monophyletic and sister to *Penola*, and so the existing treatment of these genera [1] can be maintained. *Archiborborus hirtipes* and its undescribed sister species, as well as the undescribed “daedalus” are found either in a clade with *Frutillaria* and *Penola* (Bayesian analysis of morphology, all molecular and combined analyses) or as a basal grade alongside those two genera (parsimony analyses of morphology). Two new genera will to be erected to contain them (Kits and Marshall, submitted).

Conflicting data pose some difficulty in further revising the classification of the remaining species in the subfamily. The combined parsimony analysis provides the most resolved treatment including all taxa in the subfamily, but support for most clades is very low. The type species of *Antrops* and *Archiborborus* are consistently placed in the same clade, and thus *Archiborborus* is best treated as a junior synonym of *Antrops*. Further splits of the informal groups identified here may be appropriate, but need to be justified in the context of a complete species-level revision of the subfamily (Kits and Marshall, submitted). Further data, particularly more molecular data for under-sampled clades, will undoubtedly be useful in further refining the classification of the subfamily and ensuring that generic concepts are able to stabilize.

## Supporting Information

Table S1 Specimen data for molecular exemplars. Voucher numbers for newly sequenced specimens indicate the unique identification number in the University of Guelph Insect Collection specimen database which holds full collection details; this number is also printed on individual specimen labels. Sequence data is stored in both Genbank and BOLD; the latter also includes trace files for sequences.
(DOCX)

Nexus S1 **Matrix of morphological characters.** Includes 53 characters for 128 taxa. Nexus format.
(NEX)

Nexus S2 **Matrix of molecular characters.** Includes 5 loci (2795 base pairs) for 28 taxa. Nexus format.
(NEX)

## References

[1] Kits JH, Marshall SA (2011) A revision of *Frutillaria* Richards and *Penola* Richards (Diptera: Sphaeroceridae: Archiborborinae). Zootaxa 2863: 1–34.

[2] Roháček J, Marshall SA, Norrbom AL, Buck M, Quiros DI, et al.. (2001) World catalogue of Sphaeroceridae (Diptera). Opava, Czech Republic: Slezské zemské muzeum. 414 p.

[3] Hackman W (1969) A review of the zoogeography and classification of the Sphaeroceridae (Borboridae, Diptera). Notulae Entomologicae 49: 193–210.

[4] Richards OW (1941) Sphaeroceridae (Diptera) collected by the British Graham Land Expedition, 1934–1937. British Graham Land Expedition 1934–37, Scientific Reports 1: 323–326.

[5] Richards OW (1961) Diptera (Sphaeroceridae) from South Chile. Proceedings of the Royal Entomological Society of London, Series B: Taxonomy 30: 57–68.

[6] Norrbom AL, Kim KC (1985) Systematics of *Crumomyia* Macquart and *Alloborborus* Duda (Diptera: Sphaeroceridae). Systematic Entomology 10: 167–225.

[7] Han H-Y, Ro K-E (2005) Molecular phylogeny of the superfamily Tephritoidea (Insecta: Diptera): new evidence from the mitochondrial 12S, 16S, and COII genes. Molecular Phylogenetics and Evolution 34: 416–430. 15619452 10.1016/j.ympev.2004.10.017

[8] Gibson JF, Skevington JH, Kelso S (2010) Placement of Conopidae (Diptera) within Schizophora based on mtDNA and nrDNA gene regions. Molecular Phylogenetics and Evolution 56: 91–103. 20362064 10.1016/j.ympev.2010.03.026

[9] Winkler IS, Rung A, Scheffer SJ (2010) Hennig's orphans revisited: testing morphological hypotheses in the “Opomyzoidea” (Diptera: Schizophora). Molecular Phylogenetics and Evolution 54: 746–762. 20040375 10.1016/j.ympev.2009.12.016

[10] Wiegmann BM, Trautwein MD, Winkler IS, Barr NB, Kim J-W, et al (2011) Episodic radiations in the fly tree of life. Proceedings of the National Academy of Sciences 108: 5690–5695. 10.1073/pnas.1012675108 PMC3078341 21402926

[11] McAlpine DK (2007) Review of the Borboroidini or Wombat Flies (Diptera: Heteromyzidae), with reconsideration of the status of families Heleomyzidae and Sphaeroceridae, and descriptions of femoral gland-baskets. Records of the Australian Museum 59: 1–143.

[12] Marshall SA (1996) *Tucma fritzi*, a new species in the enigmatic genus *Tucma* Mourgues-Schurter (Diptera; Sphaeroceridae; Tucminae, new subfamily). Studia Dipterologica 3: 283–288.

[13] Smith MA, Woodley NE, Janzen DH, Hallwachs W, Hebert PDN (2006) DNA barcodes reveal cryptic host-specificity within the presumed polyphagous members of a genus of parasitoid flies (Diptera: Tachinidae). Proceedings of the National Academy of Sciences 103: 3657–3662. 10.1073/pnas.0511318103 PMC1383497 16505365

[14] Ratnasingham S, Hebert PDN (2007) BOLD: The Barcode of Life Data System (http://www.barcodinglife.org). Molecular Ecology Notes 7: 355–364. 18784790 10.1111/j.1471-8286.2007.01678.x PMC1890991

[15] Simon C, Frati F, Beckenbach A, Crespi B, Liu H, et al (1994) Evolution, weighting, and phylogenetic utility of mitochondrial gene sequences and a compilation of conserved polymerase chain reaction primers. Annals of the Entomological Society of America 87: 651–701.

[16] Gibson JF, Kelso S, Jackson MD, Kits JH, Miranda GFG, et al (2011) Diptera-specific PCR-amplification primers of use in molecular phylogenetic research. Annals of the Entomological Society of America 104: 976–997.

[17] Folmer O, Black M, Hoeh W, Lutz R, Vrijenhoek R (1994) DNA primers for amplification of mitochondrial cytochrome c oxidase subunit I from diverse metazoan invertebrates. Molecular Marine Biology and Biotechnology 3: 294–297. 7881515

[18] Bertone MA, Courtney GW, Wiegmann BM (2008) Phylogenetics and temporal diversification of the earliest true flies (Insecta: Diptera) based on multiple nuclear genes. Systematic Entomology 33: 668–687.

[19] Gibson JF, Kelso S, Skevington JH (2010) Band-cutting no more: A method for the isolation and purification of target PCR bands from multiplex PCR products using new technology. Molecular Phylogenetics and Evolution 56: 1126–1128. 20460160 10.1016/j.ympev.2010.05.005

[20] Hall T (1999) BioEdit: a user-friendly biological sequence alignment editor and analysis program for Windows 95/98/NT. Nucleic Acids Symposium Series 41: 95–98.

[21] Hancock JM, Tautz D, Dover G (1988) Evolution of the secondary structures and compensatory mutations of the ribosomal RNAs of *Drosophila melanogaster* . Molecular Biology and Evolution 5: 393–414. 3136295 10.1093/oxfordjournals.molbev.a040501

[22] Will S, Reiche K, Hofacker IL, Stadler PF, Backofen R (2007) Inferring noncoding RNA families and classes by means of genome-scale structure-based clustering. PLoS Computational Biology 3: e65 doi:10.1371/journal.pcbi.0030065. 17432929 10.1371/journal.pcbi.0030065 PMC1851984

[23] Goloboff PA, Farris JS, Nixon KC (2008) TNT, a free program for phylogenetic analysis. Cladistics 24: 774–786.

[24] Swofford DL (2003) PAUP*. Phylogenetic Analysis Using Parsimony (*and Other Methods). Version 4.b10. Sunderland, Massachusetts: Sinauer Associates.

[25] Lewis PO, Holder MT, Swofford DL (2010) Phycas: software for phylogenetic analysis. Version 1.2.0. Available: http://www.phycas.org/. Accessed 2011 Apr 12.

[26] Fan Y, Wu R, Chen M-H, Kuo L, Lewis PO (2010) Choosing among partition models in Bayesian phylogenetics. Molecular Biology and Evolution 28: 523–532. 20801907 10.1093/molbev/msq224 PMC3002242

[27] Brown JM, Lemmon AR (2007) The importance of data partitioning and the utility of Bayes factors in Bayesian phylogenetics. Systematic Biology 56: 643–655. 17661232 10.1080/10635150701546249

[28] Huelsenbeck JP, Ronquist F (2001) MRBAYES: Bayesian inference of phylogenetic trees. Bioinformatics 17: 754–755. 11524383 10.1093/bioinformatics/17.8.754

[29] Ronquist F, Huelsenbeck JP (2003) MrBayes 3: Bayesian phylogenetic inference under mixed models. Bioinformatics 19: 1572–1574. 12912839 10.1093/bioinformatics/btg180

[30] Ronquist F, Teslenko M, van der Mark P, Ayres DL, Darling A, et al (2012) MrBayes 3.2: Efficient Bayesian phylogenetic inference and model choice across a large model space. Systematic Biology 61: 539–542. 22357727 10.1093/sysbio/sys029 PMC3329765

[31] Miller MA, Pfeiffer W, Schwartz T (2010) Creating the CIPRES Science Gateway for inference of large phylogenetic trees. In: Proceedings of the Gateway Computing Environments Workshop (GCE), 14 Nov 2010, New Orleans. pp. 1–8.

[32] Nylander JAA, Wilgenbusch JC, Warren DL, Swofford DL (2008) AWTY (are we there yet?): a system for graphical exploration of MCMC convergence in Bayesian phylogenetics. Bioinformatics 24: 581–583. 17766271 10.1093/bioinformatics/btm388

[33] Rambaut A, Drummond AJ (2007) Tracer version 1. 5. Available http://tree.bio.ed.ac.uk/software/tracer/. Accessed 2011 May 18.

[34] Huelsenbeck JP, Larget B, Alfaro ME (2004) Bayesian phylogenetic model selection using reversible jump Markov chain Monte Carlo. Molecular Biology and Evolution 21: 1123–1133. 15034130 10.1093/molbev/msh123

[35] Lewis PO (2001) A likelihood approach to estimating phylogeny from discrete morphological character data. Systematic Biology 50: 913–925. 12116640 10.1080/106351501753462876

[36] Aberer AJ, Krompass D, Stamatakis A (2012) Pruning rogue taxa improves phylogenetic accuracy: an efficient algorithm and webservice. Systematic Biology in press. 10.1093/sysbio/sys078 PMC3526802 22962004

[37] Marshall S (1997) *Limomyza*, a new genus of primitive Limosininae (Diptera: Sphaeroceridae), with five new species from United States, Mexico, and Central America. Proceedings of the Entomological Society of Washington 99: 279–289.

[38] Norrbom A, Kim KC (1984) The taxonomic status of *Lotophila* Lioy, with a review of *L. atra* (Meigen) (Diptera: Sphaeroceridae). Proceedings of the Entomological Society of Washington 86: 305–311.

[39] Marshall SA (1983) A revision of the genus *Aptilotus* Mik in North America (Diptera, Sphaeroceridae). Canadian Journal of Zoology 61: 1910–1924.

[40] Marshall SA, Smith I (1990) A review of the North American species of *Aptilotus*, with descriptions of new species from North America and Nepal (Diptera: Sphaeroceridae). Canadian Journal of Zoology 68: 2338–2351.

