# Supplementary material for: Phylogeny of the Archiborborinae (Diptera: Sphaeroceridae) Based on Combined Morphological and Molecular Analysis
Source: PLoS One. 2013 Jan 18;8(1):e51190. doi: 10.1371/journal.pone.0051190 (PMC3548823; doi:10.1371/journal.pone.0051190)
Supplement: Table S1 — Specimen data for molecular exemplars. Voucher numbers for newly sequenced specimens indicate the unique identification number in the University of Guelph Insect Collection specimen database which holds full collection details; this number is also printed on individual specimen labels. Sequence data is stored in both Genbank and BOLD; the latter also includes trace files for sequences. (DOCX) [file pone.0051190.s001.docx]

| Taxon | Locality | Voucher # | Genbank Accession # (12S / COI / CytB / AATS / 28S) | BOLD sample ID |
| --- | --- | --- | --- | --- |
| *Epistomyia* sp. (Heleomyzidae) | Chile: Magallanes Reg. | debu00344765 | JX887754 / JX260392 / JX887703 / JX260418 / JX887727 | DEBU0590 |
| *Pycnopota* sp. (unplaced Sphaeroceridae) | Costa Rica: Cartago | debu00344778 | JX887759 / JX260397 / JX887708 / JX260423 / JX887732 | DEBU0588 |
| *Parasphaerocera* sp. (Sphaerocerinae) | Costa Rica: Alajuela | debu00344781 | JX887758 / JX260396 / JX887707 / JX260422 / JX887731 | DEBU0587 |
| *Apteromyia* sp. (Limosininae) | Canada: Ontario | debu00344789 | JX887743 / JX260390 / JX887692 / JX260416 / JX887717 | DEBU0592 |
| *Rachispoda* sp. (Limosininae) | Canada: Ontario | N / A | HM062594 / HM062544 / HM062566 / HM062647 / HM062622 | N/A |
| *Copromyza* sp. (Copromyzinae) | USA: California | debu00344787 | JX887753 / JX260391 / JX887702 / JX260417 / JX887726 | DEBU0591 |
| *Lotophila atra* (Copromyzinae) | Canada: Ontario | debu00344790 | JX887757 / JX260395 / JX887706 / JX260421 / JX887730 | DEBU0589 |
| *Archiborborus annulatus* | Chile: Los Lagos | debu00344772 | JX887744 / JX260353 / JX887693 / JX260398 / JX887718 | DEBU0566 |
| *Archiborborus femoralis* | Chile: Magallanes | debu00344769 | JX887745 / JX260356 / JX887694 / JX260399 / JX887719 | DEBU0569 |
| *Archiborborus hirtipes* | Chile: Magallanes | debu00344766 | JX887746 / JX260358 / JX887695 / JX260400 / JX887720 | DEBU0585 |
| *Archiborborus hirtus* | Chile: Magallanes | debu00344764 | JX887747 / JX260360 / JX887696 / JX260401 / JX887721 | DEBU0579 |
| *Archiborborus maculipennis* | Chile: Magallanes | debu00344770 | JX887748 / JX260363 / JX887697 / JX260402 / JX887722 | DEBU0570 |
| *Archiborborus maximus* | Chile: Magallanes | debu00344771 | JX887749 / JX260364 / JX887698 / JX260403 / JX887723 | DEBU0575 |
| *Archiborborus orbitalis* | Bolivia: La Paz | debu00177056 | JX887750 / JX260365 / JX887699 / JX260404 / - | DEBU0580 |
| *Archiborborus quadrinotus* | Chile: Magallanes | debu00344768 | JX887751 / JX260367 / JX887700 / JX260405 / JX887724 | DEBU0568 |
| *Archiborborus simplicimanus* | Chile: Magallanes | debu00344767 | JX887752 / JX260369 / JX887701 / JX260406 / JX887725 | DEBU0571 |
| *Frutillaria calida* | Chile: Los Lagos | debu00344773 | JX887755 / JX260393 / JX887704 / JX260419 / JX887728 | DEBU0567 |
| *Frutillaria edenensis* | Chile: Los Lagos | debu00344774 | JX887756 / JX260394 / JX887705 / JX260420 / JX887729 | DEBU0573 |
| Undescribed taxa: |  |  |  |  |
| "vittatus" | Chile: Valparaiso | debu01033618 | JX887733 / JX260370 / JX887683 / JX260407 / JX887709 | DEBU0576 |
| "marensis" | Brazil: Sao Paolo | debu00344762 | JX887735 / JX260372 / JX887685 / JX260409 / JX887711 | DEBU0577 |
| "crocidosternum" | Brazil: Sao Paolo | debu00344761 | JX887736 / JX260373 / JX887686 / JX260410 / JX887712 | DEBU0578 |
| "daedalus" | Chile: Los Lagos | debu00344775 | JX887734 / JX260371 / JX887684 / JX260408 / JX887710 | DEBU0574 |
| "echinus" | Ecuador: Napo | debu00186696 | JX887737 / - / JX887687 / JX260411 / - | DEBU0582 |
| "bellavista" | Ecuador: Pichincha | debu00344486 | JX887738 / JX260376 / JX887688 / - / JX887713 | DEBU0584 |
| "auranticeps" | Ecuador: Pichincha | debu00344785 | JX887739 / JX260386 / JX887689 / JX260412 / JX887714 | DEBU0583 |
| "inbio" | Costa Rica: San José | debu00344780 | JX887740 / JX260388 / JX887690 / JX260413 / JX887715 | DEBU0586 |
| "siberia" | Bolivia: Santa Cruz | debu00127200 | JX887741 / - / - / JX260414 / - | DEBU0581 |
| "yungas" | Peru: Cuzco | debu00344784 | JX887742 / JX260389 / JX887691 / JX260415 / JX887716 | DEBU0572 |
